# Epicardial adipose tissue volume and myocardial ischemia in asymptomatic people living with diabetes: a cross-sectional study

**DOI:** 10.1186/s12933-021-01420-5

**Published:** 2021-11-24

**Authors:** Emmanuel Cosson, Minh Tuan Nguyen, Imen Rezgani, Narimane Berkane, Sara Pinto, Hélène Bihan, Sopio Tatulashvili, Malak Taher, Meriem Sal, Michael Soussan, Pierre-Yves Brillet, Paul Valensi

**Affiliations:** 1 Department of Endocrinology-Diabetology-Nutrition, Avicenne Hospital, CRNH-IdF, CINFO, AP-HP, Université Paris 13, Sorbonne Paris Cité, 125 Rue de Stalingrad, 93000 Bobigny Cedex, France; 2 Unité de Recherche Epidémiologique Nutritionnelle, UMR U1153 INSERM/U11125 INRA/CNAM/Université Paris 13, Bobigny, France; 3 Unit of Endocrinology-Diabetology-Nutrition, Jean Verdier Hospital, AP-HP, Université Paris 13, Bondy, France; 4 Unit of Diabetology, Jean Verdier Hospital, CRNH-IdF, CINFO, AP-HP, Université Paris 13, Sorbonne Paris Cité, Bondy, France; 5 Laboratoire Educations et Pratiques de Santé UR 3412, UFR Santé, Médecine, Biologie Humaine, Université Paris Sorbonne Paris Nord, 74, Rue Marcel Cachin, 93017 Bobigny Cedex, France; 6 Department of Nuclear Medicine, Avicenne Hospital, AP-HP, Bobigny, France; 7 Department of Radiology, Avicenne Hospital, AP-HP, Bobigny, France

**Keywords:** Computed tomography, Coronary artery calcification, Diabetes, Epicardial adipose tissue, Epicardial fat tissue, Myocardial ischemia, Visceral fat

## Abstract

**Background:**

Epicardial adipose tissue (EAT) is considered a novel diagnostic marker for cardiometabolic disease. This study aimed to evaluate whether EAT volume was associated with stress-induced myocardial ischemia in asymptomatic people living with diabetes—independently of confounding factors—and whether it could predict this condition.

**Methods:**

We included asymptomatic patients with diabetes and no coronary history, who had undergone both a stress a myocardial scintigraphy to diagnose myocardial ischemia, and a computed tomography to measure their coronary artery calcium (CAC) score. EAT volume was retrospectively measured from computed tomography imaging. Determinants of EAT volume and asymptomatic myocardial ischemia were evaluated.

**Results:**

The study population comprised 274 individuals, including 153 men. Mean (± standard deviation) age was 62 ± 9 years, and 243, 23 and 8 had type 2, type 1, or another type of diabetes, respectively. Mean body mass index was 30 ± 6 kg/m^2^, and mean EAT volume 96 ± 36 cm^3^. Myocardial ischemia was detected in 32 patients (11.7%). EAT volume was positively correlated with age, body mass index and triglyceridemia, but negatively correlated with HbA1c, HDL- and LDL-cholesterol levels. Furthermore, EAT volume was lower in people with retinopathy, but higher in men, in current smokers, in patients with nephropathy, those with a CAC score > 100 Agatston units, and finally in individuals with myocardial ischemia (110 ± 37 cm^3^ vs 94 ± 37 cm^3^ in those without myocardial ischemia, p < 0.05). The association between EAT volume and myocardial ischemia remained significant after adjustment for gender, diabetes duration, peripheral macrovascular disease and CAC score. We also found that area under the ROC curve analysis showed that EAT volume (AROC: 0.771 [95% confidence interval 0.683–0.858]) did not provide improved discrimination of myocardial ischemia over the following classic factors: gender, diabetes duration, peripheral macrovascular disease, retinopathy, nephropathy, smoking, atherogenic dyslipidemia, and CAC score (AROC 0.773 [0.683–0.862]).

**Conclusions:**

EAT may play a role in coronary atherosclerosis and coronary circulation in patients with diabetes. However, considering EAT volume is not a better marker for discriminating the risk of asymptomatic myocardial ischemia than classic clinical data.

## Background

Despite improved multifactorial care, diabetes is still associated with an increased risk of cardiovascular disease [1, 2]. It has been suggested that the visceral fat tissues located adjacent to the coronary arteries—especially epicardial adipose tissue (EAT)—are one of the elements linking diabetes with cardiovascular disease [3, 4] for two primary reasons: first, diabetes is accompanied by an expansion of EAT and pericardial adipose tissue [4]. Second, these tissues secrete inflammatory factors and lipid metabolites, and may be determinants of accelerated atherosclerosis [3–6].

Some studies have shown that EAT amount is associated with myocardial ischemia and/or coronary stenosis in the general population [7–11]. However, only one study to date has explored this association specifically in asymptomatic persons living with type 2 diabetes (i.e., no personal cardiovascular history or symptoms) [12]. In that study, Kim et al. showed that increased EAT thickness was an independent risk factor for coronary stenosis but not for myocardial ischemia. However, the study’s power was limited as only 100 patients were included. Detecting diabetic patients with a very high risk of asymptomatic coronary disease is clinically relevant as they could benefit from specific prevention interventions [13–15].

In this context, using a large cohort of asymptomatic patients living with diabetes, the present study aimed to evaluate whether EAT volume was associated with asymptomatic myocardial ischemia, and whether it could help discriminate patients with this condition better than classic risk markers.

## Methods

### Inclusion criteria

This observational study retrospectively recruited consecutive patients consulted between 2010 and 2019 in the diabetes clinic in Jean Verdier Hospital, in Bondy, France. Data were extracted from the hospital’s files and were anonymized.

We selected individuals with diabetes who had no personal history of coronary artery disease or associated symptom, no heart failure, a normal 12-lead resting electrocardiogram (ECG), and both a stress myocardial scintigraphy and computed tomography (CT) measurement of their coronary artery calcium (CAC) score. The latter two examinations are routinely performed to evaluate cardiovascular risk in the hospital’s Diabetology–Endocrinology–Nutrition unit [13, 14]. The same CT scans were also used to measure EAT volume.

### Data collection

Data were extracted from patients’ medical records and collected anonymously in a secure health database. For the present study, we focused on:

- General data: current tobacco consumption, diagnosed premature (before 55 years of age for men; before 65 years for women) coronary artery disease in first degree relatives.
- Medical history: routine treatments before admission, history of peripheral macrovascular disease (history of stroke, peripheral artery occlusive disease, 50% or greater stenosis measured by ultrasound examination). Hypertension and dyslipidemia were self-reported and/or inferred from prescriptions for antihypertensive and lipid-lowering agents, respectively. Additionally, we collected data to measure possible obesity (body mass index (BMI) ≥ 30 kg/m^2^). BMI was calculated using the formula: weight (kg)/height^2^ (m^2^). Weight and height were measured within 24 h of hospital admission.
- Biomarkers: HbA1c (high performance liquid chromatography variant); total and HDL-cholesterol (colorimetric assay on homogenous phase and cholesterol dosage by cholesterol oxidase), triglycerides (colorimetric assay), and LDL-cholesterol (calculated using the Friedewald formula). All these measurements were performed on plasma from fasting individuals using a Cobas 6000 analyzer (Roche diagnostics, Meylan, France). Atherogenic dyslipidemia was defined as triglycerides ≥ 2.26 mmol/L and HDL-cholesterol ≤ 0.88 mmol/L [16]. Serum creatinine was measured (colorimetry, Kone Optima, Thermolab System, Paris La Défense, France) and the glomerular filtration rate estimated (using the Chronic Kidney Disease-Epidemiology Collaboration equation). Furthermore, the urinary albumin excretion rate was measured (immunoturbidimetry, Cobas c501, Roche Diagnostics, Meylan, France), with levels between 30 and 299 mg/24 h defining microalbuminuria, and higher levels defining macroalbuminuria.
- Diabetes-related complications: retinopathy (detected by fundus photography or ophthalmoscopy), nephropathy (defined as renal failure (i.e., an estimated glomerular filtration rate < 60 mL/min) and/or micro or macroalbuminuria), neuropathy (defined as any sign or symptom of polyneuropathy), and peripheral macrovascular disease.

### Stress myocardial scintigraphy

Patients underwent a dual-isotope rest ^201^thallium/stress ^99m^Tc-sestamibi protocol or a stress/rest protocol using ^99m^Tc-sestamibi [17]. The stress test consisted in an exercise using either a calibrated bicycle ergometer or a pharmacological stress test (dipyridamole injection), or both. The former was performed when a patient was able to exercise on a bicycle ergometer and was expected to have an interpretable exercise-based ECG. The latter was performed when a patient was unable to exercise or when the exercise-based ECG stress test result was indeterminate. Asymptomatic myocardial ischemia was defined as having an abnormal ECG stress test and/or abnormal myocardial scintigraphy (i.e., defects in at least three of the 17 segmental regions).

### CT imaging

CAC scores and EAT volume were calculated using ECG-gated cardiac CT without contrast injection. All CT scans were performed with GE (Healthcare Digital, France) or Siemens (Healthineers, France) scanners. CAC scores were calculated following manufacturers’ guidelines [18] using a dedicated tool available on Picture Archiving and Communication Systems (PACS) platforms (either from Carestream Health, Rochester, NY or Philips Healthcare, Best, the Netherlands). EAT volume was quantified with the software package AW VolumeShare 7 (GE Healthcare Digital) and was measured using a semi-automatic segmentation technique on every axial slice from the thoracic inlet to the beginning of the abdomen. The software automatically measured EAT volume (in cm^3^) by summing appropriate pixels using a CT Hounsfield unit, range − 150 to − 50 HU. The software user could readjust the delimitation manually when necessary [19, 20].

### Statistical analyses

Continuous variables were expressed as means ± standard deviation and compared using one-way ANOVA or the Mann–Whitney’s U test as appropriate. No data replacement procedure was used for missing data. Pearson’s and/or Spearman’s correlations were performed to identify the parameters associated with EAT. The χ^2^ test was used to measure significant differences between the proportion of patients with or without asymptomatic myocardial ischemia.

We used the C-statistic to determine whether EAT volume and CAC score [13, 14, 21]—separately or combined—improved the prediction of the risk of myocardial ischemia over the risk predicted when using classic factors associated with asymptomatic myocardial ischemia (i.e., male gender, diabetes duration, peripheral macrovascular disease, retinopathy, nephropathy, smoking, and atherogenic dyslipidemia [16, 22, 23]). Finally, to evaluate the independent relationship between EAT volume and myocardial ischemia, we performed logistic regressions for the multivariable analyses, which included the classic variables listed above—at first separately and then all together—as well as EAT volume, the CAC score and BMI. We also evaluated the independent relationship between EAT volume and additional parameters that were associated with EAT volume, i.e. age, HbA1c, systolic blood pressure, triglycerides, HDL- and LDL-cholesterol levels. Odds ratios (OR) with 95% confidence intervals (95CI) for the risk of myocardial ischemia were calculated.

## Results

### Patient characteristics

The characteristics of the 274 included patients, including 153 men, are shown in Table 1. In summary, mean (± standard deviation) age was 62 ± 9 years, and 243, 23 and 8 had type 2, type 1, or another type of diabetes, respectively. Mean diabetes duration was 17 ± 10 years and 55.5% of the patients were treated with insulin. The percentage of obese participants was 48.1%. Mean EAT volume 96 ± 36 cm^3^ and 32 patients (11.7%) had asymptomatic myocardial ischemia.

**Table 1:** Patient characteristics according to presence of myocardial ischemia

	Available data	Total	No myocardial ischemia	Myocardial ischemia	p
		n = 274	n = 242	n = 32	
Clinical characteristics					
Age (years)	274	62.2 ± 9.5	61.9 ± 9.3	64.2 ± 10.5	0.195
Male gender	274	153 (55.8)	128 (52.9)	25 (78.1)	0.008
Body mass index (kg/m^2^)	264	30.2 ± 6.1	30.2 ± 6.1	29.7 ± 6.3	0.661
Obesity	266	128 (48.1)	115 (49.1)	13 (40.6)	0.451
Diabetes					
Type	274				0.506
Type 1		23 (8.4)	21 (8.7)	2 (6.3)	
Type 2		243 (88.7)	213 (88.0)	30 (93.8)	
Other		8 (2.9)	8 (3.3)	0 (0)	
Time since diagnosis (years)	267	17 ± 10	16 ± 9	21 ± 11	0.005
HbA1c (%)	267	8.0 ± 1.8	8.0 ± 1.9	7.9 ± 1.4	0.947
Diabetes-related treatment					
Metformin	274	209 (76.3)	182 (75.2)	27 (84.4)	0.376
Sulfonylurea	273	125 (45.8)	109 (45.2)	16 (50.0)	0.706
Alpha-glucosidase inhibitor	274	14 (5.1)	10 (4.1)	4 (12.5)	0.066
Di-peptidyl-peptidase 4 inhibitor	274	63 (23.0)	55 (22.7)	8 (25.0)	0.823
Sodium-glucose cotransporter-2 inhibitor	274	0 (0)	0 (0)	0 (0)	
Glucagon-like peptide 1 receptor agonists	274	49 (17.9)	43 (17.8)	6 (18.8)	0.811
Insulin	274	152 (55.5)	134 (55.4)	18 (56.3)	1.000
Diabetes-related complications					
Retinopathy	269	106 (39.4)	92 (38.8)	14 (43.8)	0.700
Estimated glomerular filtration rate	273				0.910
≥ 60 mL/min		228 (83.5)	202 (83.8)	26 (81.3)	
30–59 mL/min		36 (13.2)	31 (12.9)	5 (15.6)	
< 30 mL/min		9 (3.3)	8 (3.3)	1 (3.1)	
Proteinuria	268				
No		158 (59.9)	141 (59.7)	17 (53.1)	0.647
Microalbuminuria		68 (24.6)	56 (23.7)	10 (31.3)	
Macroalbuminuria		44 (16.4)	39 (16.5)	5 (15.6)	
Nephropathy	272	152 (55.9)	130 (53.9)	22 (71.0)	0.085
Neuropathy	269	179 (66.5)	154 (65.0)	25 (78.1)	0.165
Peripheral macrovascular disease	272	60 (22.1)	47 (19.6)	13 (40.6)	0.012
Additional cardiovascular risk factors					
Family history of premature CAD	236	28 (11.9)	26 (12.5)	2 (7.1)	0.545
Hypertension^a^	273	241 (88.3)	210 (87.1)	31 (96.9)	0.145
Antihypertensive treatment					0.593
Angiotensin-converting enzyme inhibitor	273	94 (34.4)	83 (34.4)	11 (34.4)	1.000
Angiotensin 2 receptor blocker	273	124 (45.4)	107 (44.4)	17 (53.1)	0.450
Beta blocker	273	54 (19.8)	46 (19.1)	8 (25.0)	0.478
Calcium channel inhibitor	273	103 (37.7)	89 (36.9)	14 (43.8)	0.446
Other	273	117 (42.9)	102 (42.3)	15 (54.9)	0.705
Dyslipidemia^a^	274	230 (83.9)	202 (87.8)	28 (12.2)	0.798
Atherogenic dyslipidemia^b^	268	17 (6.3)	16 (6.8)	1 (3.2)	0.449
Total cholesterol (mmol/L)	266	4.1 ± 1.0	4.1 ± 1.0	4.0 ± 1.1	0.525
HDL cholesterol (mmol/L)	268	1.2 ± 0.4	1.3 ± 0.4	1.1 ± 0.3	0.083
Triglycerides (mmol/L)	268	1.7 ± 1.0	1.7 ± 1.0	1.7 ± 0.9	0.083
LDL cholesterol (mmol/L)	262	2.1 ± 0.9	2.1 ± 0.9	2.1 ± 0.8	0.770
Lipid-lowering treatment					
Statin	273	201 (73.6)	179 (74.3)	22 (68.8)	0.525
Fibrates	273	10 (3.7)	7 (2.9)	3 (9.4)	0.099
Ezetimibe	273	10 (3.7)	8 (3.3)	2 (6.3)	0.331
Current smoking	266	51 (19.2)	44 (18.6)	7 (24.1)	0.459
Aspirin	272	129 (47.4)	108 (44.8)	21 (67.7)	0.021
Computed tomography					
Epicardial adipose tissue (cm^3^)	274	96 ± 36	94 ± 37	110 ± 37	0.021
Coronary artery calcium score (AU)	274	307 ± 515	272 ± 472	563 ± 722	0.003
Coronary artery calcium score > 100 AU	274	139 (50.7)	112 (46.3)	23 (71.9)	0.008

Data are given as the mean ± standard deviation or n (%)

p value: comparison between patients with and without silent myocardial ischemia

*AU* Agatston unit, *CAD* coronary artery disease

^a^Hypertension and dyslipidemia were self-reported and/or inferred from prescriptions for antihypertensive and lipid-lowering agents, respectively

^b^Atherogenic dyslipidemia was defined as triglycerides ≥ 2.26 mmol/L and HDL-cholesterol ≤ 0.88 mmol/L

### Parameters associated with EAT volume

**Fig. 1:**
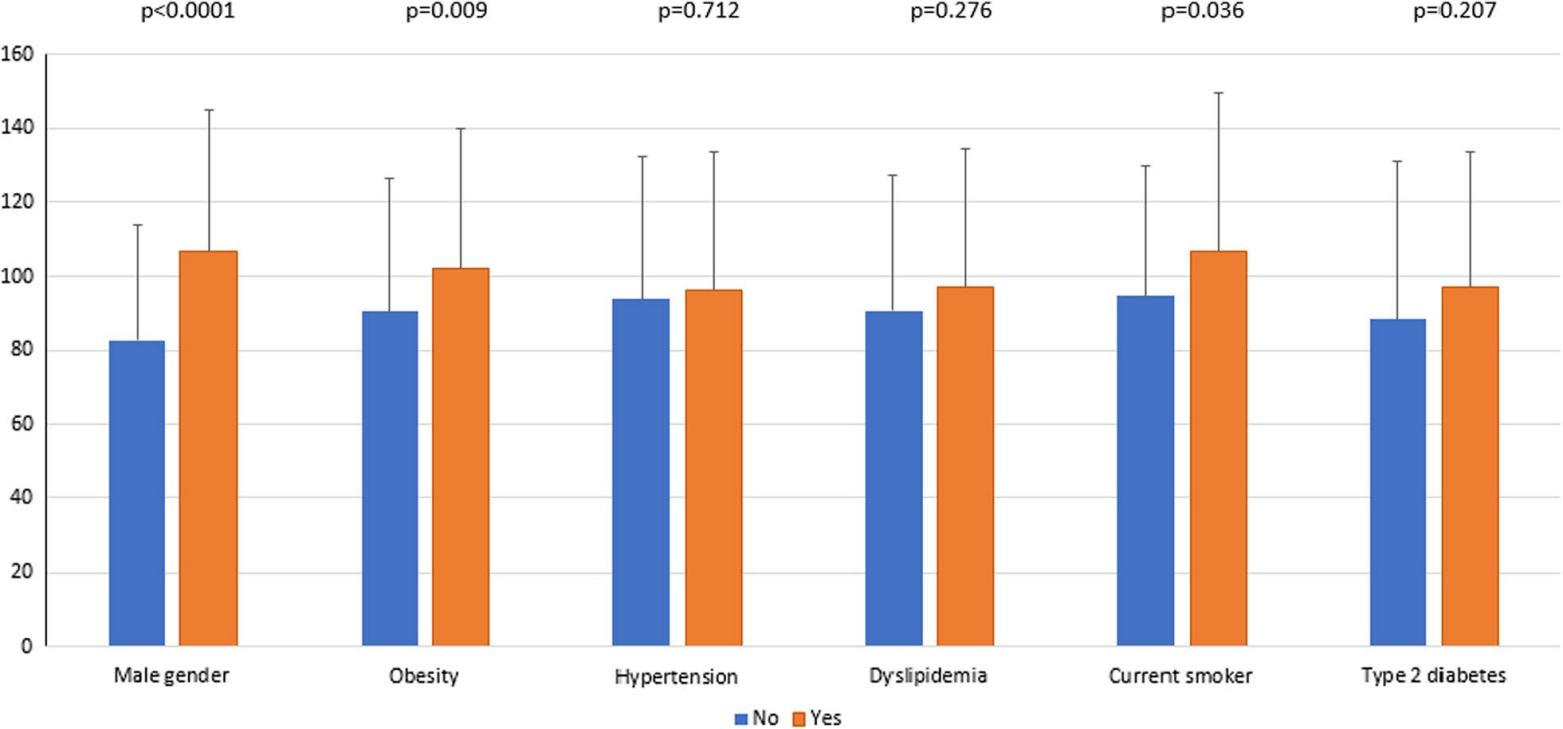
Epicardial adipose tissue volume according to cardio-vascular risk factors. Data are given as the mean ± standard deviation

**Fig. 2:**
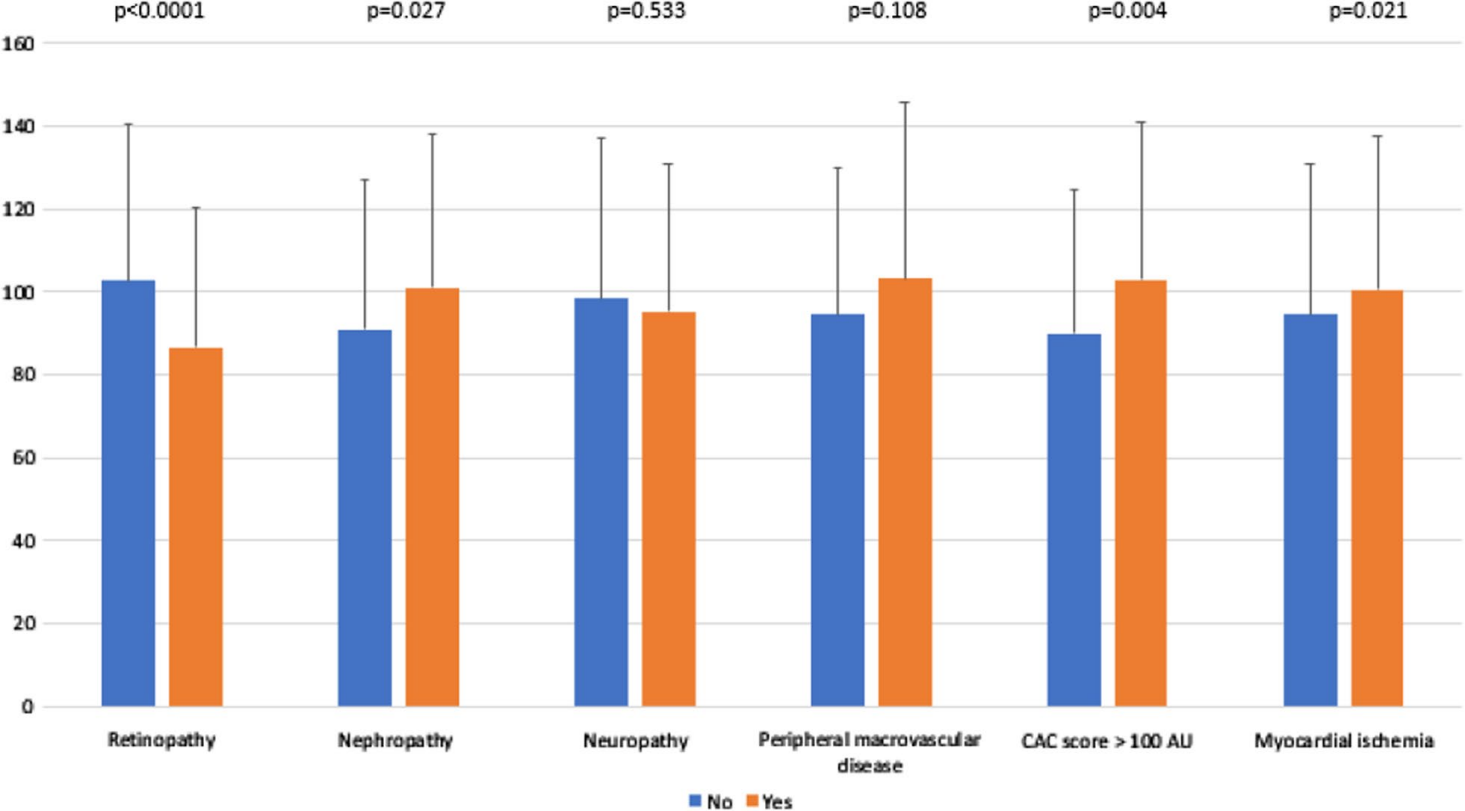
Epicardial adipose tissue volume according to diabetes-related complications. Data are given as the mean ± standard deviation; *AU* Agatston units, *CAC* coronary artery calcium

EAT volume was positively correlated with age, BMI and triglyceridemia, but negatively correlated with HbA1c, HDL- and LDL-cholesterol level (Table 2). Furthermore, it was lower in people with retinopathy than in those without (87 ± 34 vs 103 ± 38 cm^3^, p < 0.001), but higher in men than in women (107 ± 38 vs 83 ± 31 cm^3^, p < 0.01), in current smokers (107 ± 43 vs 95 ± 35 cm^3^, p < 0.05), in patients with nephropathy (101 ± 37 vs 91 ± 36 cm^3^, p < 0.05), in those with a CAC score > 100 AU (103 ± 38 vs 90 ± 34 cm^3^, p < 0.01), and finally in individuals with myocardial ischemia (94 ± 37 vs 110 ± 37 cm^3^, p < 0.05) (Figs. 1 and 2).

**Table 2:** Correlation of epicardial adipose tissue volume with quantitative data

	R	p-value
Age	0.206	< 0.001
Body mass index	0.198	< 0.001
HbA1c	− 0.134	0.028
Estimated glomerular filtration rate	− 0.041	0.503
Systolic blood pressure	0.152	0.022
Diastolic blood pressure	− 0.006	0.932
HDL cholesterol	− 0.205	< 0.001
Triglycerides	0.135	0.027
LDL cholesterol	− 0.138	0.025
Coronary artery calcium score	0.105	0.083

### Parameters associated with asymptomatic myocardial ischemia

Individuals with myocardial ischemia (versus without) were more likely to be male (OR 3.2 [95CI 1.3–7.6]), to have peripheral macrovascular disease (OR 2.8 [95 CI 1.3–6.1]), a CAC score > 100 Agatston units (AU) (OR 3.0 [95 CI 1.3–6.7]), and to be treated with aspirin (OR 2.6 [95 CI 1.2–5.7]). Furthermore, they had diabetes for a longer time (Table 1).

In the multivariable analyses, the association between EAT volume and myocardial ischemia remained statistically significant after adjustment for each of the following variables: gender, diabetes duration, peripheral macrovascular disease, and CAC score (Table 3).

**Table 3:** Association between epicardial adipose tissue volume (per 10 cm^3^ increase) and myocardial ischemia after adjustment for confounders

	Available data	Odds ratio	[95% confidence interval]	p
Crude model (no adjustment)	n = 274	1.12	[1.02–1.23]	0.023
Adjustment for body mass index (kg/m^2^)	n = 264	0.97	[0.91–1.04]	0.365
Adjustment for gender	n = 274	3.18	[1.33–7.63]	0.010
Adjustment for diabetes duration (years)	n = 267	1.64	[1.16–2.32]	0.005
Adjustment for peripheral macrovascular disease	n = 272	2.59	[1.18–5.68]	0.018
Adjustment for retinopathy	n = 269	1.53	[0.70–3.35]	0.281
Adjustment for nephropathy	n = 272	1.89	[0.83–4.32]	0.126
Adjustment for atherogenic dyslipidemia	n = 268	0.41	[0.50–3.24]	0.384
Adjustment for smoking	n = 266	1.17	[0.46–3.00]	0.743
Adjustment for coronary artery calcium score (Agatston unit)	n = 274	1.10	[1.00–1.22]	0.011
Adjustment for age (years)	n = 274	1.0	[1.0–1.0]	0.354
Adjustment for triglyceridemia (mmol/L)	n = 268	1.0	[0.7–1.5]	0.848
Adjustment for LDL-cholesterol (mmol/L)	n = 262	1.0	[0.6–1.6]	0.974
Adjustment for HDL-cholesterol (mmol/L)	n = 268	0.4	[0.1–1.4]	0.150
Adjustment for HbA1c (%)	n = 267	1.0	[0.8–1.3]	0.819
Adjustment for systolic blood pressure (mmHg)	n = 227	1.0	[1.0–1.0]	0.929
Adjustment for classic risk factors for asymptomatic myocardial ischemia^a^ and coronary artery calcium score	n = 247	1.08	[0.97–1.22]	0.130

^a^Gender, diabetes duration, peripheral macrovascular disease, retinopathy, nephropathy, smoking, atherogenic dyslipidemia

**Fig. 3:**
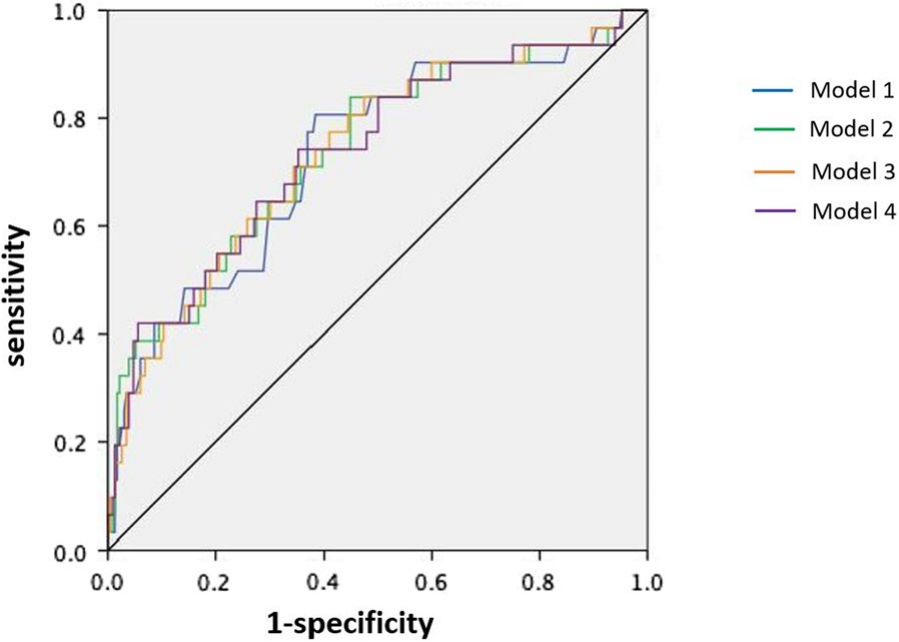
Area under the curve to predict asymptomatic myocardial ischemia. Model 1 (classic risk factors: male gender, diabetes duration, peripheral macrovascular disease, retinopathy, nephropathy, atherogenic dyslipidemia, smoking): area under the ROC curve (AROC [95% confidence interval]) 0.770 [0.680–0.860]). Model 2 (Model 1 + epicardial adipose tissue (EAT) volume): AROC 0.767 [0.679–0;856]. Model 3 (Model 1 + coronary artery calcium score (CAC) score): AROC 0.773 [0.683–0.862]. Model 4 (Model 1 + EAT volume + CAC score): AROC 0.771 [0.683–0.858]

Additionally, neither EAT volume nor CAC score—separately or combined—were better at discriminating the risk of myocardial ischemia over classic risk factors (Fig. 3). Specifically, the areas under the ROC curve (AROC [95CI]) were 0.770 [0.680–0.860] for classic risk factors, 0.767 [0.679–0,856] for classic risk factors and EAT volume, 0.773 [0.683–0.862] for classic risk factors and CAC score, and finally 0.771 [0.683–0.858] for classic risk factors and both EAT volume and CAC score.

## Discussion

Our cohort study results show that EAT volume was significantly associated with stress-induced myocardial ischemia in asymptomatic people with diabetes, and that this association remained significant after controlling for gender, diabetes duration, peripheral macrovascular disease, and CAC score. However, EAT volume did not improve discrimination of ischemia over these classic risk factors.

In contrast, in their Korean cohort, Kim et al*.* did not report a significant association between EAT thickness and asymptomatic myocardial ischemia or infarction (with vs. without: 12.8 ± 2.1 vs 11.7 ± 2.3 mm, respectively, p = 0.11) [12]. This discrepancy with our results may be due to better statistical power in our study than theirs (274 vs 100 participants, respectively), the different type of EAT measurement (volume vs thickness), the different method used to screen for myocardial ischemia (scintigraphy vs magnetic resonance acquired during adenosine stress and at rest), and different patient profiles (ethnicity, BMI 30 vs 25 kg/m^2^, diabetes duration 17 vs 8 years, and HbA1c level 8 vs 7%) [12].

There are arguments for a causal relationship between EAT and myocardial ischemia. First, increased EAT volume/thickness has been associated with other markers of subclinical atherosclerosis patients with diabetes including high CAC score [19], arterial stiffness [24] and cardiac dysfunction [3]. Second, prospective studies have shown that high EAT volume/thickness is predictive of a higher incidence of cardiovascular events in the general population [25] and in patients with type 2 diabetes [26, 27]. Third, the positive association between EAT and myocardial ischemia may reflect pathophysiological effects of EAT on coronary circulation. This hypothesis is supported by other studies reporting a similar association [7–11]. However, inclusion criteria in those studies differed from ours as they considered only between 7% [11] and 36% [9] of patients with diabetes, persons in secondary prevention [9], and/or persons with chest pain [7–11]. More specifically, several pathophysiological pathways may be involved in the association. First, EAT volume has been reported to be higher in patients with coronary stenoses [9, 10, 12, 28] and is associated with plaque vulnerability, which may contribute to acute coronary syndrome [29]. It also distinguishes patients with *vs* without myocardial infarction [30]. Second, it has been suggested that EAT is an important source of energy for the myocardium during periods of increased energy demand through lipolysis and fat oxidation, leading to putative lipotoxicity in cardiomyocytes and disruption of fatty acid beta oxidation [3]. Third, in patients without significant coronary stenosis, ischemia may result from functional disorders, such as abnormal coronary reserve and endothelial dysfunction [31–33]. It has been shown that abnormal increases in EAT volume are proinflammatory and that EAT secretes vasoactive factors that regulate coronary endothelial function and facilitate free fatty acid influx [3–6]. However, some studies have suggested that no association exists between EAT and microvascular function [34] or coronary vasomotor dysfunction in patients with diabetes [35] (although the same studies did find such associations in individuals without diabetes).

The association between EAT volume and ischemia in our study population may be partially due to confounding factors. EAT volume and asymptomatic myocardial ischemia share similar risk factors, such as male gender, age, diabetes duration, lipid disorders, nephropathy, peripheral macrovascular disease, and a high CAC score [3, 12, 16, 19, 22, 23]. We found that EAT was associated with myocardial ischemia independently of gender, diabetes duration, peripheral macrovascular disease and CAC score, but not independently of the other confounders listed above. This means that control of cardiovascular risk factors, including BMI, lipid, glucose, blood pressure and smoking may explain a higher risk of both myocardial ischemia and higher EAT volume. Specific mechanistic studies are therefore needed to fully understand how EAT could foster ischemia in the diabetic population. Finally, our results showed that EAT volume did not improve discrimination of predicted risk of asymptomatic myocardial ischemia over classic factors, suggesting that the screening strategies currently proposed [13, 14] would not be improved if EAT volume were measured concurrently with CAC score.

Our study has several limitations. First, it was observational in design, which prevented us from being able to draw conclusions about causal relationships between EAT volume and myocardial ischemia. Second, we only included patients who had been admitted to our hospital department and who had both a myocardial scintigraphy and a CAC score measurement. Therefore, our results may not be representative of all patients with diabetes. Third, we did not have data on ethnicity, which is a determinant of EAT volume in the diabetic population [19]. Fourth, we did not include an invasive angiography to assess potential coronary stenosis in patients with myocardial ischemia. Fifth, we explored global but not regional EAT volume in the heart [10, 11, 36], and EAT volume but not its density. Having said that, density was not associated with myocardial ischemia in a previous study [11]. Finally, we did not have any data on EAT function [3, 37], such as inflammation or brown fat activity.

The main strength of our study is that we measured EAT and not pericardial (or total cardiac) adipose tissue. EAT lies between the myocardium and the visceral layer of the pericardium and is different from pericardial fat, which is located externally to the myocardium. As no fascia separates EAT from the myocardium, they are in direct contact [3–6]. To date, EAT is the only type of cardiac adipose tissue which has been observed to predict incident cardiovascular events in people with type 2 diabetes [26]. Furthermore, we applied a robust methodology—CT acquisition and assessment following standard methods—and used specific cardiac software to automatically quantify EAT. CT scans are considered the gold standard for EAT as, unlike echography, they measure EAT volume not thickness [5, 27].

## Conclusions

We showed that EAT volume was significantly higher in asymptomatic individuals with myocardial ischemia—specifically stress-induced myocardial ischemia—who had diabetes, and that this association remained significant after adjustment for gender, diabetes duration, peripheral macrovascular disease and CAC score. Finally, EAT volume did not improve the prediction of the risk of ischemia over these classic risk factors in this population.

## Data Availability

Data for the present analysis can be provided from the first author on reasonable request.

## Abbreviations

95CI: 95% Confidence interval
AROC: Area under the operating curve
BMI: Body mass index
CAC: Coronary artery calcium
CT: Computed tomography
EAT: Epicardial adipose tissue
ECG: Electrocardiogram
PACS: Picture Archiving and Communication Systems
OR: Odds ratio
